# Sword Bean (*Canavalia gladiata*) Pod Exerts Anti-Allergic and Anti-Inflammatory Effects through Modulation of Th1/Th2 Cell Differentiation

**DOI:** 10.3390/nu14142853

**Published:** 2022-07-12

**Authors:** Kyung-A Hwang, Yu Jin Hwang, Hye-Jeong Hwang, Sang Hoon Lee, Young Jun Kim

**Affiliations:** 1 Department of Agrofood Resources, National Institute of Agricultural Sciences, Rural Development Administration, Wanju-gun 55365, Korea; yjhwang1022@korea.kr (Y.J.H.); hjh1027@korea.kr (H.-J.H.); 2 Department of Food and Biotechnology, Korea University, Sejong 30019, Korea; t9592359@korea.ac.kr

**Keywords:** sword bean pod, *Canavalia gladiata*, anti-allergic, anti-inflammatory

## Abstract

Allergy is an immunoglobulin E (IgE)-mediated process, and its incidence and prevalence have increased worldwide in recent years. Therapeutic agents for allergic diseases are continuously being developed, but side effects follow when used for a long-term use. Therefore, treatments based on natural products that are safe for the body are urgently required. Sword bean (*Canavalia gladiata*) pod (SBP) has been traditionally used to treat inflammatory diseases, but there is still no scientific basis for its anti-allergic effect. Accordingly, this study investigates the anti-allergic effect and its mechanism of SBP in vitro and in vivo. SBP reduced the nitric oxide production and decreased mRNA and protein expression of inflammatory mediates (inducible nitric oxide synthase (iNOS) and cyclooxygenase-2 (COX-2)), and inhibited the phosphorylation of nuclear factor kappa B (NF-κB), a major signaling molecule in the inflammatory response. Additionally, SBP extract treatment inhibited phosphatidylinositol-3-kinase/mammalian target of rapamycin (PI3K/mTOR) signaling activity to further inhibit degranulation and allergy mediator generation and control the balance of Th1/Th2 cells, which can induce an allergic reaction when disrupted. Furthermore, the SBP extract exhibited anti-allergic effects in anti-dinitrophenyl IgE-induced RBL-2H3 cells and ovalbumin-treated mice. These findings have potential clinical implications for the treatment as well as prevention of allergic diseases.

## 1. Introduction

Allergic diseases are immediate-type allergic reactions mediated by immunoglobin E (IgE) [1]. IgE and antigen bind specifically to the surface receptor (FcεRI) induce degranulation of mast cells, and histamine and cytokines present in the granules are secreted out of the cell. Through this process, atopic dermatitis, asthma, and rhinitis are induced via smooth muscle contraction, vascular permeability enhancement, and inflammatory response promotion [2,3,4]. The incidence of these allergic diseases is rapidly increasing worldwide due to complex causes such as westernized diets, environmental pollution, adult diseases, and genetic factors. In particular, the prevalence is reported to be extremely high in children under the age of 10, which is a pressing health problem that needs a solution [5].

Additionally, allergic reactions are related to a reduction in the production of anti-inflammatory cytokines (interferon gamma (IFN-γ) and interleukin-12 (IL-12)) and an increase in the production of proinflammatory cytokines (interleukin-4 (IL-4) and interleukin-13 (IL-13)), which play an important part in the differentiation of T cells into T helper (Th) 1 and Th2 cells [6,7]. Each cytokine helps the T cells differentiation by activating a certain type of transcription factor. For Th1 cells, IL-12 and IFN-γ activate the differentiation of naive CD4+ T cells by increasing the expression of the transcription factors signal transducer and activator of transcription 1 (STAT1) and T-box transcription factor TBX21 (T-bet). In Th2 cell differentiation, expression of the transcription factors STAT6 and GATA-3 is increased and activated by IL-4 [8]. When the balance of Th1/Th2 cells is disrupted, an allergic reaction is induced. Several studies are underway to improve allergic and inflammatory reactions by regulating the activity of Th1/Th2 cells.

The Th1/Th2 cell balance is a major factor in the induction of allergic diseases, and T cells rely on mammalian target of rapamycin (mTOR) signaling to maintain immune and metabolic signals in appropriate states [9]. mTOR is a serine/threonine-specific protein kinase belonging to the phosphoinositide 3-kinases (PI3K) family that plays a central role in regulating cell growth and metabolism [10,11,12]. When a foreign antigen is recognized in the body, the expression of mTOR, which activates CD4+ T cells to induce Th1, Th2, or Th17 cell differentiation, is increased. This response promotes lymphocyte immune regulation and airway smooth muscle growth via the PI3K/mTOR pathway during mast cell degranulation and under asthmatic conditions [13,14]. As such, the ultimate treatment for improving immune regulation and asthma could be to maintain the balance of Th1/Th2 cells, which depends on PI3K/mTOR activity.

Anti-allergic drugs of various components are being developed to suppress these mechanisms. Histamine, a representative agent that causes allergy, was discovered in the 1920s. Many drugs were developed that focus on blocking histamine activity (e.g., Azelastine, Levocabastine, Cyproheptadin, etc.). However, antihistamines provide partial relief from symptoms and do not block other allergy mediators. In addition, leukotriene and prostaglandins are released from mast cells at the same time as histamine is secreted. Therefore, various drugs such as leukotriene inhibitors and mast cell stabilizers that can inhibit these substances have been developed to improve allergy symptoms that reduce quality of life [15]. However, the effect is temporary, and long-term use causes side effects such as orthostatic hypotension, dementia, arrhythmias, etc. [16,17], so no drug has been found that can calm all allergic reactions. In order to fundamentally solve these problems, the development of anti-allergic agents and supplements using plant-derived natural products such as licorice and green tea, which have no toxicity or side effects, is attractive [18,19,20,21].

Sword bean (SB; *Canavalia gladiata*) is a leguminous annual plant native to Asia and Africa and traditionally used as a food and medicine [22,23]. SB can be used on all parts such as seeds, pods, stems, and roots, and contains a large number of functional components including flavonoids, tannins, saponins, terpenoids, and steroids. The nutritional components of dried soybeans and pods consist of general components such as protein, fat, and moisture, as well as various vitamins and minerals, and the contents are shown in Supplementary Materials Table S1. Recently, various functional activities of SB such as anti-obesity [24], antioxidant [25], anti-inflammatory [26], hematopoietic expansion improvement [27], hepatoprotective, and anti-angiogenic effects [28] have been reported. However, scientific research using SB pods (SBP) has not been conducted except for anti-inflammation and anti-obesity, and the mechanism is not yet clearly defined. [29,30].

Therefore, this study aims to investigate the anti-allergic and anti-inflammatory effects of SBP using both in vitro and in vivo systems. Furthermore, we want to confirm the potential of SBP as a major anti-allergy therapeutic agent through Th1/Th2 cell balance by regulating the activity of the PI3K/mTOR mechanism. Through this study, it is to provide basic data for the future development of health-functional foods using this natural product.

## 2. Materials and Methods

### 2.1. Materials

Dulbecco’s Modified Eagle Medium (DMEM), phosphate-buffered saline (PBS), and penicillin-streptomycin antibiotics (P/S) were supplied by Gibco (Gaithersburg, MD, USA). Fetal bovine serum (FBS) was obtained from GenDEPOT (Barker, TX, USA). Primary antibodies and secondary antibodies were purchased from Abcam (Cambridge, MA, USA). Lipopolysaccharide (LPS), ovalbumin (OVA) from chicken egg, aluminum hydroxide (Alum), anti-dinitrophenyl (DNP) IgE, DNP-human serum albumin (HSA), 3-(4,5-dimethylthiazol-2-yl)-2,5-diphenyltetrazolium bromide (MTT), and dimethyl sulfoxide (DMSO) were purchased from Sigma-Aldrich (St. Louis, MO, USA).

### 2.2. Preparation of the Extract

SBP was purchased from Hwasun, Jeollanam-do, South Korea. Before extracting the raw material, SBP was washed with water (to remove impurities) and then minced, dried at 50 °C for 8 h (Cheil Machinery, Icheon, Korea), and grinded (IKA, M20, Staufen, Germany). The SBP powder added 30% ethanol and was stirred at 80 °C for 8 h. After the primary extraction, secondary extraction was performed at 80 °C for 4 h by adding 30% ethanol. The obtained extract was filtered, and the filtrate was concentrated using a vacuum rotary evaporator (Doo Young High Technology, Seoul, Korea). Then, maltodextrin was added in the same amount as the solid content of the concentrate, followed by stirring at 95 °C for 1 h. The stirred concentrate was recovered as a dry powder using a hot air dryer and stored at −20 °C before use.

### 2.3. Cell Culture

Raw264.7 as mouse macrophage cell and RBL-2H3 as rat mast cell were obtained from the Korean Cell Line Bank (Seoul, Korea). Raw264.7 and RBL-2H3 cells were maintained in DMEM contained supplemented with 10% FBS and 1% P/S at 37 °C in a 5% carbon dioxide (CO_2_) incubator. Culture medium was changed every two days, and cell density was maintained at 80–90% during subculture.

### 2.4. Cell Viability

Raw264.7 and RBL-2H3 cells were seeded in a 96-well-plate. Cells were incubated with SBP extract (20, 40, 100, and 200 μg/mL) for 24 h. MTT reagent was added to each well. After 4 h incubation, the medium was removed, and formazan were dissolved with DMSO. The absorbance was subsequently measured at 540 nm. Results were expressed as a percentage versus that in the untreated group.

### 2.5. Nitric Oxide (NO) Production

Raw264.7 cells were dispensed into a 96-well plate, treated with the SBP extract in various concentrations, and then treated with LPS (1 μg/mL). After 24 h incubation, supernatant was recovered, and the amount of NO production was measured using the Griess reagent (Promega, Madison, WI, USA). The amount of NO produced was calculated using sodium nitrite as a standard; a standard curve was created, and the absorbance value was substituted into the calculation formula to calculate the amount of NO produced.

### 2.6. Quantitative Reverse Transcriptase-PCR (qRT–PCR)

mRNA expression of inflammatory genes (*iNOS, COX-2, IL-4*, *IL-13, IFN-γ*, and *IL-12,*) and PI3K/mTOR mechanism genes (*PI3K*, *Akt*, *mTOR*) were measured. Raw264.7 were treated with LPS and SBP extract. RBL-2H3 cells were treated with anti-DNP IgE (200 ng/mL), with the SBP extract, and then with DNP-HAS (20 ng/mL) for 1 h. The total RNA was extracted from the cultured cells using the RNeasy plus Mini Kit obtained from Qiagen (Valencia, CA, USA). After that, 500 ng of total RNA was synthesized as cDNA using reverse transcriptase (Promega, Madison, WI, USA). qRT-PCR was performed using SYBR Green master mix (Qiagen, Valencia, CA, USA) on the Rotor Gene Q (Qiagen, Valencia, CA, USA). Gene expression levels were normalized using *GAPDH* and relative gene expression was determined by the comparative CT (2^−^^△△^^Ct^) method. The primer sequences are listed in Table 1.

### 2.7. Western Blot Analysis

The LPS (1 μg/mL) or anti-DNP IgE (200 ng/mL) and SBP extract-treated cells were lysed in a lysis buffer (50 mM Tris-hydrochloride (HCl), 150 mM sodium chloride, 0.5% Triton X-100, 0.5% NP-40, 0.1% sodium deoxycholate, and 1 mM EDTA) for 40 min on the ice. Then, the supernatant was collected through centrifugation (12,000× *g*, 4 °C, 20 min). Proteins were separated via 4–20% sodium dodecyl-sulfate polyacrylamide gel electrophoresis (SDS-PAGE) and then transferred onto a polyvinylidene fluoride (PVDF) membrane. The membrane was blocked with skim milk for 1 h and then incubated overnight at 4 °C with appropriate primary antibodies (iNOS, COX-2, STAT1, p-STAT1, T-bet, interferon regulatory factor 1 (IRF1), STAT6, p-STAT6, GATA binding protein 3 (GATA3), c-maf, and β-actin). After incubation, the cells were washed with 1× TBST and incubated with the secondary antibody for 1 h. Enhanced chemiluminescence was used to develop protein bands, and the signal was detected using a Chemi-doc image detector (Bio-Rad, Hercules, CA, USA).

### 2.8. Degranulation in RBL-2H3 Mast Cells

RBL-2H3 cells were seeded in 96-well plates, incubated for 2 h, treated with anti-DNP IgE (200 ng/mL), and then again incubated overnight. The samples were then washed twice with PBS and cultured for 24 h; then, they were added with DNP-BSA (20 ng/mL) for 1 h, and the supernatants were recovered. The culture supernatant was placed on a plate with 4-methylumbelliferly-N-acetyl-β-D-glucosaminide and allowed to react at 37 °C for 1 h. Then, the amount of β-hexosaminidase produced was measured using a colorimetric method [31], and the results were expressed as a percentage.

### 2.9. Experimental Animals

BALB/c mice (male, 6 weeks old) were purchased from Samtako (Seoul, Korea). Animals were provided with water and food ad libitum and maintained in a controlled environment at 22 ± 2 °C for 12 h under light-dark cycle. After acclimatizing the animals to the environment for one week, they were divided into five groups (eight animals per group) as follows (Table 2): (1) normal group, no sensitization with PBS; (2) negative control (NC) group, OVA/Alum-sensitized + 200 mg/kg maltodextrin; (3) positive control (PC) group, OVA/Alum-sensitized + 0.5 mg/kg dexamethasone; (4) SBP low (SBP100) group, OVA/Alum-sensitized + 100 mg/kg SBP; and (5) SBP high (SBP200) group, OVA/Alum-sensitized + 200 mg/kg SBP via oral administration (p.o.) for four weeks. After four weeks, mice were sacrificed and the blood plasma, tissues were collected. Then, a tracheostomy was performed, 1 mL PBS was injected into the bronchial tubes, and the bronchoalveolar lavage fluid (BALF) was recovered. The liver and BALF were immediately removed and stored at −80 °C until use. This study was conducted with approval from the Institutional Animal Care and Use Committee of Korea University (KUIACUC-2021-0061).

### 2.10. Sensitization and Challenge

Mice acclimatized for one week were intraperitoneally injected with 50 μg OVA and 2 mg Alum in a 1:1 ratio at weeks 1 and 3 for allergy induction. After the second intraperitoneal injection, a 5% OVA intranasal challenge was performed for three days to establish an animal model of OVA/Alum-sensitized hypersensitivity immunity.

### 2.11. Measurement of Hepatotoxicity in the Plasma

The activities of alanine aminotransferase (ALT), aspartate aminotransferase (AST) in the blood and the activities of glutathione (GSH), and glutathione peroxidase (GPx) in the liver collected after sacrifice of mice were measured using a colorimetric kit (Abcam, Cambridge, MA, USA).

### 2.12. Measurement of IgE and Histamine Production

Total IgE levels and histamine concentration in the plasma and the cell supernatant were performed using an ELISA kit (Abcam, Cambridge, MA, USA), according to the manufacturer’s instructions.

### 2.13. Measurement of Cytokine Levels in the BALF and Plasma

The levels of inflammatory cytokines (IFN-γ, IL-4, and interleukin-5 (IL-5)) in the BALF and plasma of SBP extract-treated OVA-induced mice were measured using the ELISA kit (Abcam, Cambridge, MA, USA), according to the manufacturer’s instructions.

### 2.14. Statistical Analysis

In this study, all data were expressed as mean ± standard error of the mean (SEM). Data was statistically evaluated by Student’s *t*-test. (SPSS version 25.0, Chicago, IL, USA). Differences were considered significant at *p* < 0.05.

## 3. Results

### 3.1. Cytotoxicity of the SBP Extract on Raw264.7 and RBL-2H3 Cells

**Figure 1:**
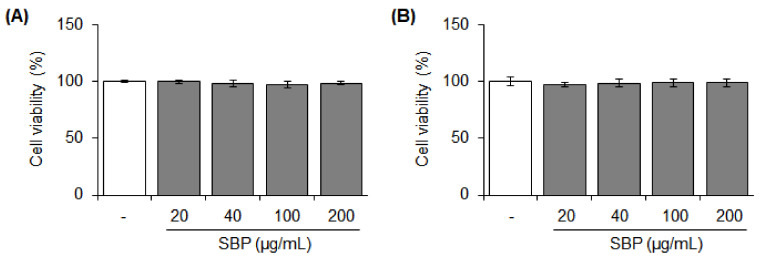
Cytotoxic effects of sword bean pod (SBP) extract on (**A**) Raw264.7 and (**B**) RBL−2H3 cells. Data are expressed as mean ± SEM. SEM, standard error of the mean; SBP, sword bean pod.

Stimulation and toxicity to cells did not occur at any tested concentrations of the SBP extract (Figure 1). Considering the use of this extract for the development of future health-functional food materials, 200 μg/mL concentration of the SBP extract was deemed safe and suitable and was used in the subsequent experiments.

### 3.2. Effect of the SBP Extract on NO Production in LPS-Induced Raw264.7 Cells

**Figure 2:**
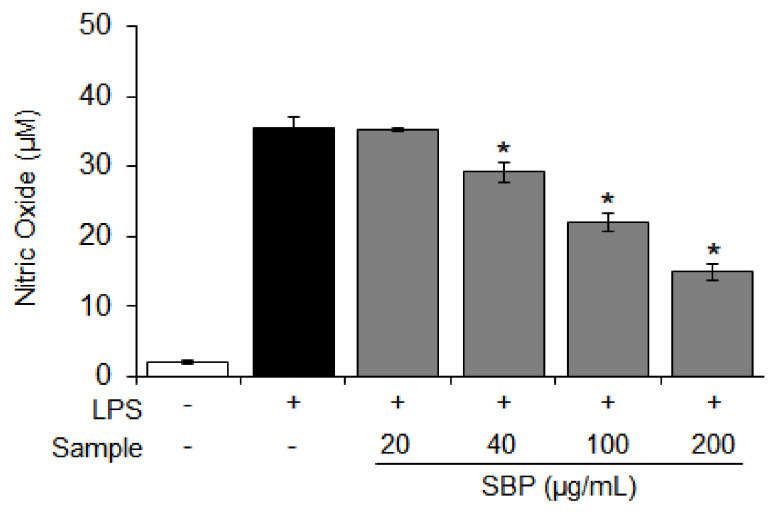
Effect of the SBP extract on production of nitric oxide in Raw264.7 cells. Data are expressed as mean ± SEM. Significantly different from the LPS−treated group (* *p* < 0.05). SBP, sword bean pod; LPS, lipopolysaccharide.

The NO is a major mediator of the inflammatory response, and excessive production of NO activates the expression of COX-2 [32]. Considering these findings, we investigated the inhibition of NO production when Raw264.7 cells induced by LPS were treated with the SBP extract. The amount of NO produced in the inflammation-induced group was increased (35.5 μM) compared to that in the control group, and the amount of NO production was reduced in a dose-dependent manner (* *p* < 0.05) upon treatment with the SBP extract (Figure 2).

### 3.3. Effects of the SBP Extract on iNOS and COX-2 mRNA and Protein Expression in LPS-induced Raw264.7 Cells

**Figure 3:**
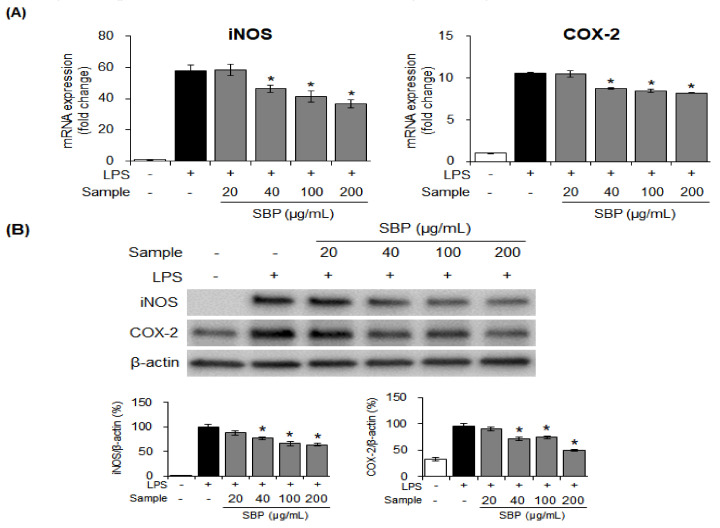
Effects of the SBP extract on mRNA and protein expression of iNOS and COX−2 in Raw264.7 cells. (**A**) mRNA and (**B**) protein expression. Data are expressed as mean ± SEM. Significantly different from the LPS−treated group (* *p* < 0.05). SBP, sword bean pod; LPS, lipopolysaccharide; iNOS, inducible nitric oxide synthase; COX-2, cyclooxygenase-2.

To determine whether the SBP extract had an inhibitory effect on NO production because of the regulation of iNOS and COX-2, the mRNA and protein expression of iNOS and COX-2 was measured. Treatment with the SBP extract significantly reduced iNOS and COX-2 mRNA and protein expression in a concentration-dependent manner (* *p* < 0.05). This indicated that the SBP extract could regulate the inflammatory reaction by suppressing the expression of inflammation-mediator genes (Figure 3).

### 3.4. Effect of the SBP Extract on NF-κB Signaling in LPS-Induced Raw264.7 Cells

**Figure 4:**
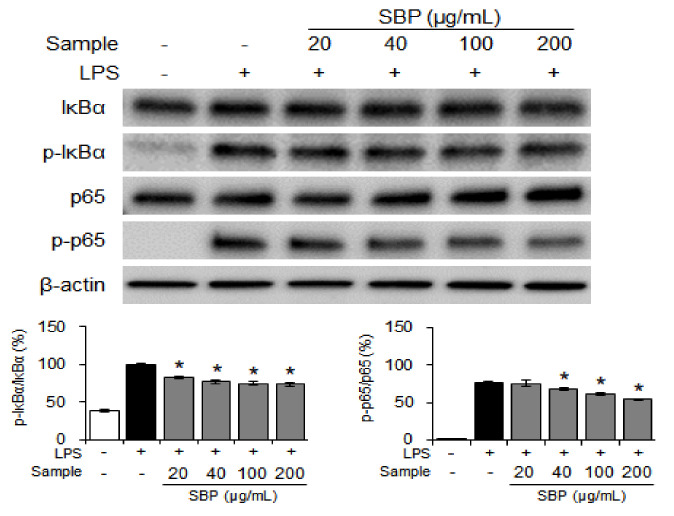
Effects of the SBP extract on NF−κB protein expression in Raw264.7 cells. Data are expressed as mean ± SEM. Significantly different from the LPS-treated group (* *p* < 0.05). SBP, sword bean pod; LPS, lipopolysaccharide. NF−κB, nuclear factor kappa B; IκBα, inhibitor of the transcriptional factor NF-κB, p-, phosphorylated.

Under normal conditions, NF-κB binds to inhibitor of the transcriptional factor NF-κB (IκBα) and maintains an inactive state. When stimulated by LPS, cytokines, and reactive oxygen species, NF-κB is activated via phosphorylation of IκBα, resulting in the increased expression of inflammatory cytokines, COX-2, and iNOS, and may lead to inflammatory and degenerative diseases [33,34,35,36]. Therefore, the effect of SBP extract on NF-κB transcriptional activity was studied using western blot analysis. The SBP extract dose-dependently inhibited the expression of p-IκBα and p-p65 after activation by LPS (Figure 4).

### 3.5. Effect of SBP Extract on the β-Hexosaminidase Release and Histamine Degranulation Stimulated by Anti-DNP IgE in RBL-2H3 Cells

**Figure 5:**
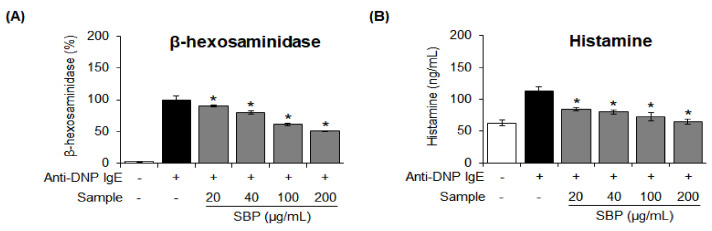
Effect of the SBP extract on β−hexosaminidase release and histamine production in RBL−2H3 cells. (**A**) β−hexosaminidase activity and (**B**) histamine production. Data are expressed as mean ± SEM. Significantly different from the LPS−treated group (* *p* < 0.05). SBP, sword bean pod Anti-DNP, anti-dinitrophenyl; IgE, immunoglobulin E.

β-hexosaminidase is a key marker of degranulation of mast cells, and its secretion is increased during the allergic inflammatory response [37]. We investigated the effect of SBP extract on β-hexosaminidase degranulation; the results are shown in Figure 5A. When allergen-induced RBL-2H3 cells were treated with 20, 40, 100, and 200 μg/mL of SBP extract, the inhibitory effect on β-hexosaminidase secretion was evident at the maximum concentration of the extract. 

Histamine, a major cause of allergic diseases, is mainly stored in basophils and mast cells. The secretion of histamine is elevated during allergic reactions and anaphylaxis [38]. In this study, the amount of histamine produced in allergenic cells was increased to 109.5 μg/mL compared to that in the control group. Nevertheless, the extent of histamine production was significantly (* *p* < 0.05) suppressed after treatment with the SBP extract (Figure 5B).

### 3.6. Effects of SBP Extract on the mRNA Expression of Pro- and Anti-Inflammatory Cytokines Stimulated by Anti-DNP IgE in RBL-2H3 Cells

Cytokines are mediate signal transduction between cells. When mast cells are activated, the pro-inflammatory cytokines such as IL-4 and IL-13, secreted from Th2 cells, activate B cells and generate an allergic reaction by producing IgE. Conversely, the anti-inflammatory cytokines, IFN-γ and IL-12, secreted from Th1 cells suppress the differentiation of T lymphocytes and reduce the production of Th2 cell-secreted cytokines, thereby alleviating allergic reactions by suppressing IgE production [39].

**Figure 6:**
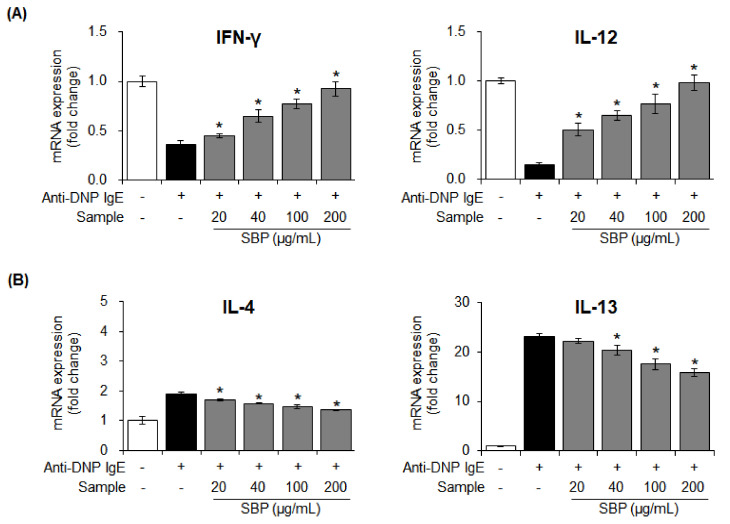
Effects of the SBP extract on mRNA expression of pro− (**B**) and anti−inflammatory (**A**) cytokine in RBL−2H3 cells. Data are expressed as mean ± SEM. Significantly different from the LPS−treated group (* *p* < 0.05). SBP, sword bean pod; IFN-γ, interferon gamma; Il-12, interleukin-12; IL-4, interleukin-4; IL-13, interleukin-13.

The mRNA expression of pro- and anti-inflammatory cytokines secreted from Th1/2 cells after the treatment of activated mast cells with the SBP extract was measured with Real-Time Quantitative Reverse Transcription PCR (qRT-PCR). In the allergy-induced group, the mRNA expression of *IFN-γ* and *IL-12* was reduced, and the expression of *IL-4* and *IL-13* was significantly increased after treatment with anti-DNP IgE. However, treatment with SBP extract led to the recovery of the expression of anti-inflammatory cytokines (IFN-γ, IL-12) to the level in the control group (* *p* < 0.05). Contrastingly, the extent of pro-inflammatory cytokine secretion (IL-4, IL-13) was significantly (* *p* < 0.05) reduced upon treatment with the SBP extract (Figure 6).

### 3.7. Effects of SBP Extract on the mRNA Expression of PI3K/mTOR Signaling Factors Stimulated by Anti-DNP IgE in RBL-2H3 Cells

**Figure 7:**
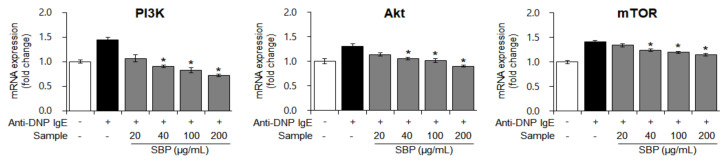
Effect of the SBP extract on mRNA expression of PI3K/mTOR in RBL−2H3 cells. Data are expressed as mean ± SEM. Significantly different from the LPS-treated group (* *p* < 0.05). SBP, sword bean pod. PI3K, phosphoinositide 3-kinases; Akt, protein kinase B; mTOR, mammalian target of rapamycin.

When a foreign antigen invades the body, a normal or allergic state is established via immune regulation, mainly by the activity of T cells. The growth of T cells, which are mainly involved in immunity, is regulated by the activation of PI3K/mTOR signaling, and degranulation of mast cells activates PI3K/mTOR sub-factors [40]. Based on this theory, we measured the gene expression of PI3K/mTOR pathway factors in allergen-sensitized mast cells (treated with anti-DNP IgE) treated with the SBP extract. The mRNA expression of *PI3K*, *Akt*, and *mTOR* was increased by allergic stimulation; nevertheless, with SBP extract treatment, this effect decreased in a dose-dependent manner (* *p* < 0.05) (Figure 7). We speculate that the SBP extract is effective in regulating T-cell growth and immune activity, because the highest concentration of the SBP extract restored the gene expression of PI3K/mTOR pathway factors to a level like that in the non-allergic normal group.

### 3.8. Effect of SBP Extract on the Protein Expression of Th1/Th2 Differentiation Transcription Factors in RBL-2H3 Cells Stimulated by Anti-DNP IgE

**Figure 8:**
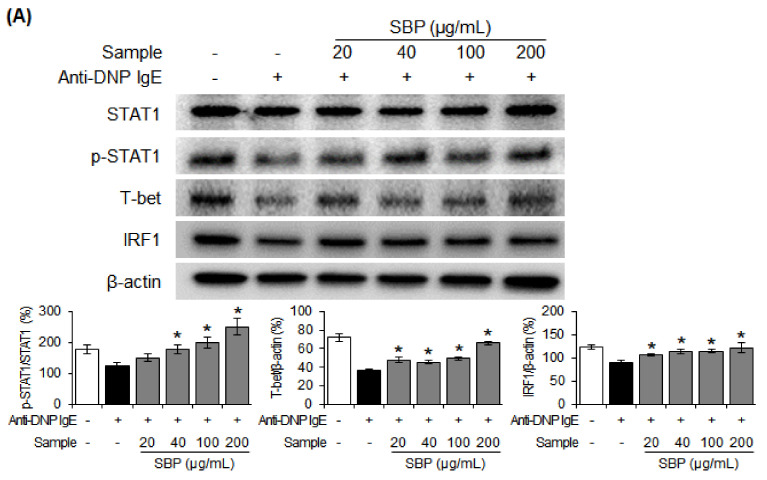

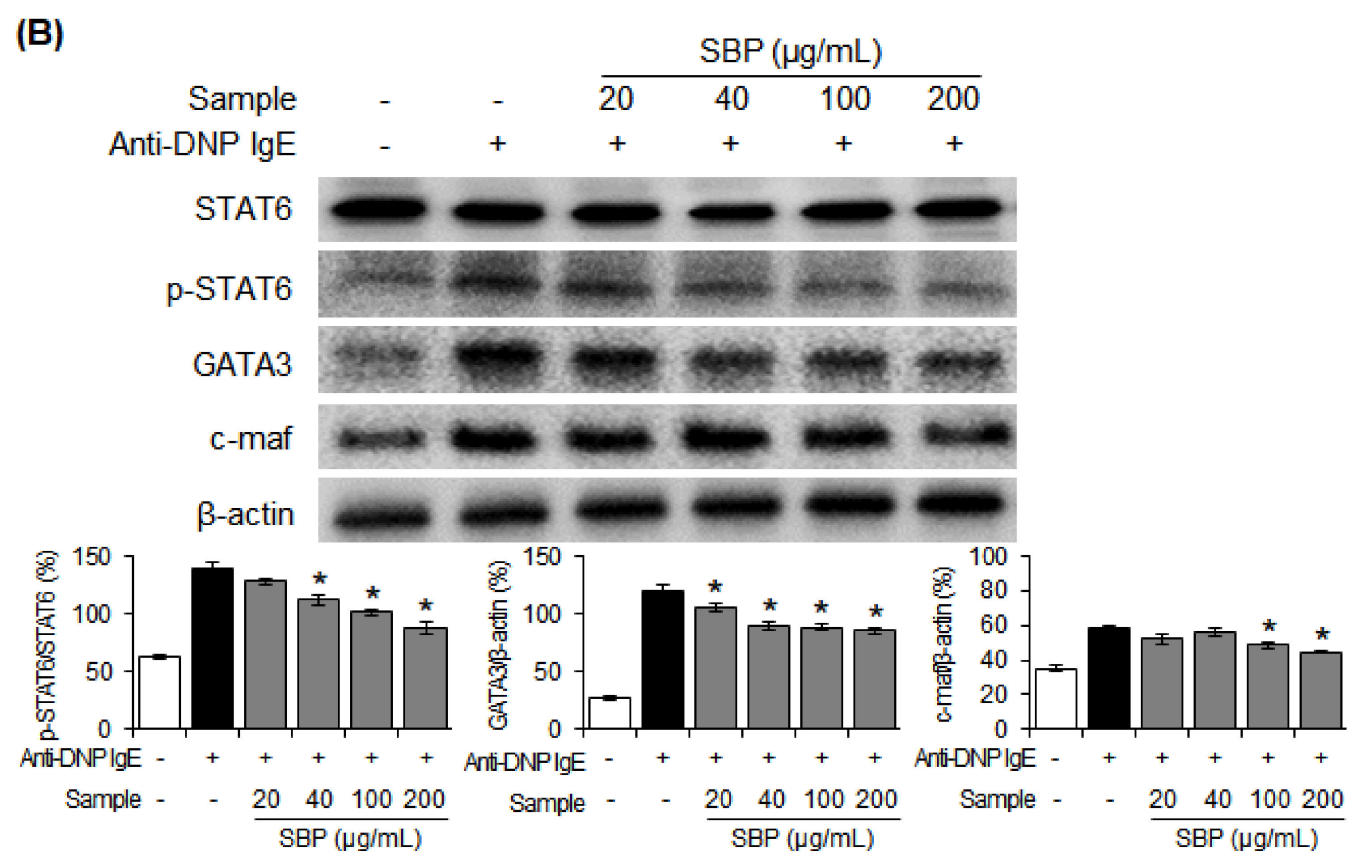
Effect of SBP extract on the protein expression of transcription factors related to the differentiation of (**A**) Th1 and (**B**) Th2 cells in anti−DNP IgE-sensitized RBL−2H3 cells. Data are expressed as mean ± SEM. Significantly different from the LPS−treated group (* *p* < 0.05). SBP, sword bean pod. STAT1, signal transducer and activator of transcription; T-bet, T-box transcription factor TBX21; IRF1, interferon regulatory factor 1; GATA3, GATA binding protein 3; c-maf, transcription factor.

After confirming that the SBP extract can regulate the mRNA expression of anti-inflammatory and pro-inflammatory cytokines and suppress that of the PI3K/mTOR signaling factors that regulate T-cell differentiation, we finally investigated the mechanism of Th1/Th2 cell differentiation using western blotting. The protein expression of Th1 cell differentiation-related transcription factors p-STAT1, T-bet, and IRF1 was reduced, while the expression of Th2 cell differentiation transcription factors p-STAT6, GATA3, and c-maf was increased in the allergic group. The expression of p-STAT1, T-bet, and IRF1 increased with increasing concentrations of the SBP extract in the allergic group, while the increase in the expression of p-STAT6, GATA3, and c-maf was suppressed (Figure 8).

### 3.9. Effects of the SBP Extract on Body and Tissue Weights of OVA/Alum-Sensitized Mice

Table 3 shows the effects of SBP extract on the body and tissue weights of experimental animals. There was no significant difference in body weights among all the groups. This was also the case for the weights of all tissues except the kidney, for which a significant (* *p* < 0.05) difference was confirmed between the NC and normal groups. Other than that, on kidney weight, the SBP extract did not show any significant effect on the weights of tissues under the present experimental conditions.

### 3.10. Hepatotoxicity of the SBP Extract in OVA/Alum-Sensitized Mice

When AST and ALT levels were measured to study the hepatotoxicity of the SBP extract, no significant differences were observed among the groups. In addition, GSH concentration was confirmed in the liver tissue. The GSH content of the NC group was 4.451 μM, a reduction of approximately 57% compared to that in the normal group (10.25 μM). Similar to the GSH results, GPx concentration decreased in the NC group and increased after administration of the SBP extract, but this increase was not statistically significant (Table 4). These results indicate that the SBP extract is not toxic.

### 3.11. Effects of the SBP Extract on Allergy Mediators in OVA/Alum-Sensitized Mice

**Figure 9:**
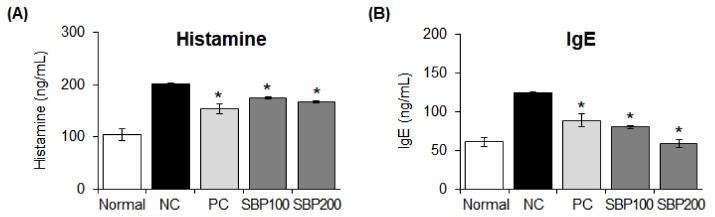
Effect of SBP extract on allergy mediator production in OVA/Alum−sensitized mice. Levels of (**A**) histamine and (**B**) IgE were measured in the plasma of OVA/Alum−sensitized mice treated with the SBP extract for four weeks. Data are expressed as mean ± SEM. Significantly different from the NC group (* *p* < 0.05). SBP, sword bean pod; NC, negative control; PC, positive control.

We measured plasma IgE and histamine concentrations in mice administered the SBP extract and found that these concentrations were significantly (* *p* < 0.05) increased in the NC group compared to those in the normal group. Allergy was induced, as evident by the increase in the concentrations of the two compounds, and treatment with the SBP extract reduced histamine and IgE concentrations similarly or more effectively than in the drug administration group (PC) (* *p* < 0.05) (Figure 9).

### 3.12. Effect of the SBP Extract on PI3K/mTOR Signaling in OVA/Alum-Sensitized Mice

**Figure 10:**
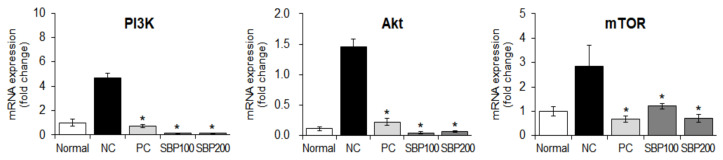
Effects of SBP extract on the mRNA expression of PI3K/mTOR signaling factors in OVA/Alum−sensitized mice liver tissue. Data are expressed as mean ± SEM. Significantly different from the NC group (* *p* < 0.05). SBP, sword bean pod; NC, negative control; PC, positive control.

Next, we investigated the mechanisms underlying the effects of SBP extract on PI3K, Akt, and mTOR expression in OVA/Alum-induced mice. The NC group induced with OVA/Alum showed increased *PI3K*, *Akt*, and *mTOR* expression compared with the normal group without any treatment, and this effect was reversed in the drug-treated (PC) and SBP extract-administered groups (* *p* < 0.05) (Figure 10).

### 3.13. Effect of the SBP Extract on Cytokine Production in OVA/Alum-Sensitized Mice

T cells are important immune cells that regulate allergic reactions and are differentiated into Th1 and Th2 depending on the type of cytokine they encounter. T-cell differentiation-regulating cytokines suppress the allergic reaction by maintaining the homeostasis of the immune response, and when this balance is disturbed, an allergic reaction is induced [41].

**Figure 11:**
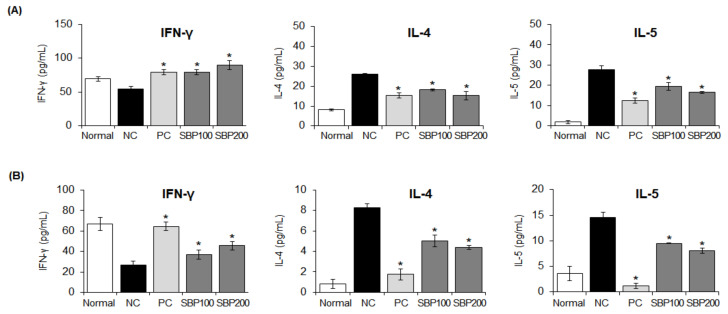
Effect of the SBP extract on T cell−differentiation cytokine production in OVA/Alum−sensitized mice. (**A**) bronchoalveolar fluid and (**B**) plasma. Data are expressed as mean ± SEM. Significantly different from the NC group (* *p* < 0.05). SBP, sword bean pod; NC, negative control; PC, positive control.

Therefore, the effect of SBP extract on T-cell differentiation-regulating cytokine production was investigated. Cytokine production was measured in the BALF and plasma. IFN-γ (Th1 cell-differentiation cytokine) production increased in a concentration-dependent manner (* *p* < 0.05) in the SBP extract group compared with that in the NC group. IL-4 and IL-5 (Th2 cell-differentiation cytokines) production was reduced (* *p* < 0.05) (Figure 11).

## 4. Discussion

With increasing interest of individuals in a healthy lifestyle, effective prevention and treatment methods for diseases are also attracting attention. In particular, the number of consumers willing to maintain their health and avoid the risk of diseases through the consumption of health-functional foods prepared from natural materials that can help avoid the side effects of therapeutic agents and maximize their beneficial effect is steadily increasing [42,43,44].

Therefore, in this study, the anti-allergic effect and action mechanism of SBP, a plant-derived natural material, were investigated for the eventual treatment of allergic diseases, which deteriorate the quality of life of modern people. In vitro, we confirmed the anti-inflammatory effect of SBP extract through its inhibitory effects on phosphorylation of the inflammatory mediators iNOS and COX-2 and the signaling factor NF-kB, a transcription factor regulating the inflammatory response. In addition, the production of histamine and β-hexosaminidase was inhibited in a concentration-dependent manner with SBP extract treatment. It has been reported that these anti-inflammatory and anti-allergic effects are regulated by Th1/2 cells [45]. Therefore, to elucidate the molecular basis for these observations, we investigated the Th1/Th2 cell-differentiation signaling mechanisms involved in inflammation and allergic responses. 

The types of differentiation of T cells are divided according to the types of cytokines they encounter. IFN-γ and IL-12, which differentiate Th1 cells, suppress the differentiation of T lymphocytes to reduce the production of Th2 cell-secreting cytokines and reduce IgE production, thereby alleviating allergic reactions. The IL-4 and IL-13 cytokines produce IgE through increased secretion and action of B cells, which differentiates Th2 cells and causes allergic reactions [46]. Similarly, our study results showed that an expression of anti-inflammatory cytokines IFN-γ and IL-12 was reduced while that of pro-inflammatory cytokines IL-4 and IL-13 was increased upon allergic and inflammatory responses. SBP extract treatment recovered the expression of these cytokines to the level in the normal group. This finding is consistent with reports that it is associated with increased production of IL-4, IL-5, and IL-13 and decreased production of IFN-γ, TNF-α, and IL-12 during inflammatory and allergic reactions [47]. In addition, cytokines are regulated by various transcription factors that differentiate T cells, thereby maintaining the Th1/Th2 cell balance. Specifically, T-bet is a Th1 transcription factor that regulates the expression of IFN-γ, and GATA3 induces STAT6 activation and the production of pro-inflammatory cytokines IL-4 and IL-5 while inhibiting Th1 expression [20]. GATA3 expression is inhibited by IFN-γ and IL-12. Taken together, we hypothesize that the SBP extract activated T-bet and suppressed GATA3 by increasing the expression of IFN-γ, and the decrease in GATA3 activity inhibited IL-4 and IL-5 production via inactivation of STAT6. Through these results, we predict that the SBP extract can modulate the immune response by regulating the differentiation of T cells. 

SBP suppressed the gene expression of the PI3K/mTOR mechanism of action that regulates T cell differentiation and growth and inflammatory response, thereby restoring the Th1/Th2 cell balance from the allergy state to the normal state and improving the biomarker of allergic symptoms. This is a conclusion similar to the study results in that mTOR activation in asthma was positively correlated with loss of Th17/Treg and Th1/Th2 balance [48].

Through the results of this study, the relationship between the immune regulation mechanisms of SBP was confirmed. First, STATs directly or indirectly regulate NF-κB activity [49]. Interestingly, STAT1 modulates the NF-κB-mediated signaling by interacting with the overlapping region of the p300 cofactor of NF-κB and the p65 subunit. Tyrosine-phosphorylated STAT6 directly binds to NF-κB in vitro and in vivo and activates the inflammatory cytokine IL-4 [29]. In addition, OVA activates the TLR4 pathway and its target, NF-κB, to exacerbate inflammation through increased secretion of Th2-secreted pro-inflammatory cytokines. Hence, inhibition of NF-κB can reduce the asthma by OVA. Accordingly, Helala et al. reported that the inhibitory signaling pathway of TLR4/NF-κB is a promising target for asthma treatment [50]. Second, Akt and mTOR factors are activated via the phosphorylation of PI3K under asthmatic conditions. In asthma, mTOR activation is positively correlated with loss of Th17/Treg and Th1/Th2 balance [48]. We also demonstrated that mTOR inhibitors effectively reduced the growth of airway-proliferating cells and disturbed the Th17/Treg and Th1/Th2 imbalance.

Our previous findings have shown that SBP includes many phenolic compounds with anti-inflammatory and anti-allergic activities. Sinapic acid and pyrogallol are found in SBP [30]. Nakano et al. reported that pyrogallol can regulate the inflammatory response in bronchial epithelial cells, and that pyrogallol extracted from Awatea exerts anti-allergic effects by suppressing nasal symptoms and the expression of IL-9 gene expression [51]. In addition, sinapic acid inhibits the expression of iNOS, COX-2, and cytokines through NF-κB inactivation, thereby providing a mechanism for its anti-inflammatory effect [52]. Additionally, methyl gallate, gallic acid, and ellagic acid among other phenolic compounds in SBP have stronger antioxidant and anti-inflammatory effects than soybeans.

Finally, it is thought that SBP exhibits anti-inflammatory and anti-allergic effects by functional ingredients such as various phenolic compounds. In addition, the anti-inflammatory and anti-allergic effect leads to the regulation of cytokine secretion through the regulation of Th1/Th2 cell differentiation activity, which acts as a major key to regulate immune activity. In particular, it is thought that SBP corrects the balance of Th1/Th2 cells and alleviates the symptoms by inhibiting PI3K/mTOR activity, a mediating mechanism of action of allergic diseases such as asthma caused by hypersensitivity immunity.

## 5. Conclusions

In this study, SBP appears to exert anti-inflammatory and anti-allergic effects through inhibition of NF-kB signaling (possibly via components such as phenolic compounds) and regulation of STAT activation. The signaling pathway PI3K/mTOR, which is considered to be important in the treatment of asthma, is also involved in the effects of SBP, which suggests that SBP is a promising potential target for the development of therapeutic agents.

As the current results have only been confirmed in vitro and in vivo, if the anti-allergic and anti-inflammatory effects of SBP can be confirmed in additional human clinical trials, this can be useful not only as a natural ingredient in health-functional foods but also as a natural agent for developing allergy treatment strategies.

## Figures and Tables

**Table 1:** The primer sequence used for qRT-PCR.

Target Gene	Primer	Sequence (5′→3′)
*mGADPH*	Forward	GTT GTC TCC TGC GAC TTC A
	Reverse	GGT GGT CCA GGG TTT CTT A
*miNOS*	Forward	GGC AGC CTG TGA GAC CTT TG
	Reverse	GCA TTG GAA GTG AAG CGT TT
*mCOX-2*	Forward	TTG CTG TAC AAG CAG TGG CAA AGG
	Reverse	TGG GAG GCA CTT GCA TTG CAT TGA
*mPI3K*	Forward	GAA GTT GCT CTA CCC AGT GTC C
	Reverse	GAT AGC CGT TCT TTT CAT TTG G
*mAkt*	Forward	ACT CAT TCC AGA CCC ACG AC
	Reverse	AGC CCG AAG TCC GTT ATC TT
*mmTOR*	Forward	TGT GAA CGG AAC ATA CGA CC
	Reverse	TTG CTT GCC CAT CAG AGT CAG
*rGADPH*	Forward	CCA CAG TCC ATG CCA TCA C
	Reverse	TCC ACC ACC CTG TTG CTG TA-
*rIFN-γ*	Forward	AAT GGC AAC ATC AGG TCG GCC ATC ACT
	Reverse	GCT GTG TGT GTC ACA GAA GTC TCG AAC TC
*rIL-12*	Forward	GGA GAG ACT ATC AAG ATA GT
	Reverse	ATG GTC AGT AGA CTT TTA CA
*rIL-4*	Forward	CGA TGA TGC ACT TGC AGA AA
	Reverse	TGG AAA TTG GGG TAG GAA GG-
*rIL-13*	Forward	AGC ACA GAA AGC ATG ATC CG
	Reverse	GTT TGC TAC GAC GTG CGC TA
*rPI3K*	Forward	AAC ACA GAA GAC CAA TAC TC
	Reverse	TTC GCC ATC TAC CAC TAC
*rAkt*	Forward	GTG GCA AGA TGT GTA TGA G
	Reverse	CTG GCT GAG TAG GAG AAC
*rmTOR*	Forward	GGT GGA CGA GCT CTT TGT CA
	Reverse	AGG AGC CCT AAC ACT CGG AT

qRT-PCR, Real-Time Quantitative Reverse Transcription PCR. *mGADPH*, *miNOS*, *mCOX-2*, *mPI3K*, *mAkt*, *mmTOR*, *rGADPH*, *rIFN-γ*, *rIL-12*, *rIL-4*, *rIL-13*, *rPI3K*, *rAkt* and *rmTOR* are gene names.

**Table 2:** Animal experimental groups and treatments.

Group (*n* = 8)	Treatment (mg/kg)	Condition
Normal	PBS	None
NC	Maltodextrin (200 mg/kg)	OVA/Alum
PC	Dexamethasone (0.5 mg/kg)	
SBP100	SBP low (100 mg/kg)	
SBP200	SBP high (200 mg/kg)	

NC: negative control, PC: positive control, PBS: phosphate buffered saline, OVA/Alum: ovalbumin/aluminum hydroxide; SBP: sword bean pod.

**Table 3:** Body and tissue weights of OVA/Alum−sensitized mice.

Group	Body (g)	Liver (g)	Spleen (g)	Kidney (g)	Heart (g)	Lung (g)
Normal	26.71 ± 0.47	1.509 ± 0.025 *	0.101 ± 0.003 *	0.452 ± 0.024	0.146 ± 0.004 *	0.307 ± 0.010 *
NC	25.72 ± 0.37	1.269 ± 0.037	0.112 ± 0.003	0.396 ± 0.019	0.131 ± 0.003	0.356 ± 0.015
PC	24.32 ± 0.24	1.063 ± 0.049 *	0.045 ± 0.003 *	0.335 ± 0.012	0.118 ± 0.004 *	0.225 ± 0.025 *
SBP100	25.26 ± 0.32	1.138 ± 0.054	0.107 ± 0.005	0.326 ± 0.005 *	0.119 ± 0.004	0.338 ± 0.010
SBP200	25.19 ± 0.22	1.192 ± 0.015	0.113 ± 0.004	0.340 ± 0.010 *	0.119 ± 0.001 *	0.330 ± 0.010

Data are expressed as mean ± SEM. * *p* < 0.05, compared with the NC group. OVA: ovalbumin, Alum: aluminum hydroxide, NC: negative control, PC: positive control, SBP: sword bean pod.

**Table 4:** AST and ALT levels in the plasma and antioxidant enzyme levels in the livers of OVA/Alum-sensitized mice.

Group	AST (mU/mL)	ALT (mU/mL)	GSH (μM)	GPx (mU/mL)
Normal	109.5 ± 5.142	61.52 ± 11.95	10.25 ± 1.750 *	672.3 ± 44.96 *
NC	118.4 ± 5.860	73.56 ± 8.811	4.451 ± 1.477	450.6 ± 104.2
PC	110.6 ± 8.071	61.94 ± 7.618	12.17 ± 0.941 *	651.1 ± 31.52 *
SBP100	109.7 ± 5.329	64.72 ± 8.760	9.414 ± 1.149 *	478.1 ± 47.16
SBP200	108.0 ± 7.734	63.48 ± 6.197	10.98 ± 1.070 *	548.6 ± 52.85

Data are expressed as mean ± SEM. * *p* < 0.05, compared with the NC group. AST: aspartate aminotransferase, ALT: alanine aminotransferase, OVA; ovalbumin, Alum: aluminum hydroxide, GSH; glutathione, GPx: glutathione peroxidase, NC: negative control, PC: positive control, SBP: sword bean pod.

## Data Availability

Not applicable.

## Supplementary Materials

The following supporting information can be downloaded at: https://www.mdpi.com/article/10.3390/nu14142853/s1, Table S1: Nutritional composition of sword bean and pod.
