# Supplementary material for: Sword Bean (Canavalia gladiata) Pod Exerts Anti-Allergic and Anti-Inflammatory Effects through Modulation of Th1/Th2 Cell Differentiation
Source: Nutrients. 2022 Jul 12;14(14):2853. doi: 10.3390/nu14142853 (PMC9322724; doi:10.3390/nu14142853)
Supplement: Supplementary file 1 [file nutrients-14-02853-s001.zip › nutrients-1784989-supplementary.pdf]

Table S1. Nutritional composition of sword bean and pod.

Ingredients	SB	SBP
**General component (g/100 g)**		
Protein	26.3	13.8
Lipid	1	9.8
Moisture	12	7.5
**Mineral (mg/100 g)**		
Calcium	84	324.5
Phosphorus	353	253.5
Iron	3.7	26.4
Potassium	1296	1549.7
Sodium	5	11.4
**Vitamin (mg/100 g)**		
β-carotene	–	0.57
Vitamin B1	0.54	0.48
Vitamin B2	0.25	0.38
Vitamin C	0	27.15
Niacin	2	3.03

This data is presented based on 100 g of dry raw material. SB, sword bean; SBP, sword bean pod.
